# Ki67 and LSD1 Expression in Testicular Germ Cell Tumors Is Not Associated with Patient Outcome: Investigation Using a Digital Pathology Algorithm

**DOI:** 10.3390/life12020264

**Published:** 2022-02-10

**Authors:** Beatriz Chaves Lourenço, Catarina Guimarães-Teixeira, Bianca C. T. Flores, Vera Miranda-Gonçalves, Rita Guimarães, Mariana Cantante, Paula Lopes, Isaac Braga, Joaquina Maurício, Carmen Jerónimo, Rui Henrique, João Lobo

**Affiliations:** 1 Department of Pathology, Portuguese Oncology Institute of Porto (IPOP), 4200-072 Porto, Portugal; up201605750@icbas.up.pt (B.C.L.); rita.guimaraes@ipoporto.min-saude.pt (R.G.); mferreira@ipoporto.min-saude.pt (M.C.); ana.ambrosio@ipoporto.min-saude.pt (P.L.); 2 Cancer Biology and Epigenetics Group, Research Center of IPO Porto (CI-IPOP)/RISE@CI-IPOP (Health Research Network), Portuguese Oncology Institute of Porto (IPO Porto)/Porto Comprehensive Cancer Center (Porto.CCC), 4200-072 Porto, Portugal; catarina.guimaraes.teixeira@ipoporto.min-saude.pt (C.G.-T.); bianca.troncarelli@ipoporto.min-saude.pt (B.C.T.F.); Vera.Miranda.Goncalves@ipoporto.min-saude.pt (V.M.-G.); carmenjeronimo@ipoporto.min-saude.pt (C.J.); 3 Department of Pathology and Molecular Immunology, ICBAS–School of Medicine and Biomedical Sciences, University of Porto (ICBAS-UP), Rua Jorge Viterbo Ferreira 228, 4050-513 Porto, Portugal; 4 Department of Urology, Portuguese Oncology Institute of Porto (IPOP), 4200-072 Porto, Portugal; isaac.braga@ipoporto.min-saude.pt; 5 Department of Medical Oncology, Portuguese Oncology Institute of Porto (IPOP), 4200-072 Porto, Portugal; jmauricio@ipoporto.min-saude.pt

**Keywords:** germ cell tumors, testicular cancer, biomarkers, histopathology, prognosis, Ki67, LSD1

## Abstract

TGCTs represent a model of curable disease afflicting especially young men. Defining tumor biological characteristics is crucial to increase current knowledge and tailor the best clinical management. Ki67, a potential prognostic marker, still exhibits heterogenous associations with patient outcomes, thus bringing the need of corroboration with larger cohorts in clinical practice. LSD1, an epigenetic enzyme, represents a future target for epigenetic drugs that may lower treatment-associated morbidity. This study aimed to assess Ki67/LSD1 immunoexpression across all TGCT histological subtypes and correlate it with clinicopathological features. Results were compared with an in silico analysis of the TCGA database. Immunohistochemistry for Ki67 and LSD1 was carried out in a cohort of 157 TGCT tumor samples and assessed using a digital pathology algorithm. LSD1 protein expression was explored in TGCT cell lines, including ATRA-differentiated clones. There was a significant positive correlation between Ki67 and LSD1 H-scores (r_s_ = 0.182, *p* = 0.037). Ki67 positivity percentage and H-score were significantly higher in non-seminomas (*p* = 0.0316 and 0.0113, respectively). Expression was not significantly different according to clinicopathological features, including stage, IGCCCG prognosis-based system, or relapse/progression-free survival, which was corroborated by in silico analysis. Our study, making use of digital image analysis, does not confirm the utility of these biomarkers in a daily practice cohort. Although not affecting patient outcome in our cohort, LSD1 is expressed overall in TGCTs, suggesting sensitivity to LSD1 inhibitors.

## 1. Introduction

Although rare, representing only 1% of male malignancies worldwide, testicular germ cell tumors (TGCTs) are the most common solid testicular neoplasms (95%) in men between the ages of 20 and 34 years, presenting a rising global incidence over the past decades [1,2]. In particular, type II TGCTs, the most frequent testicular neoplasms, exhibit a striking complex and heterogenous histology. Their common precursor is germ cell neoplasia in situ (GCNIS), and they are divided into seminomas (SE) and non-seminomas (NS). The latter comprise a complex array of subtypes that include embryonal carcinoma (EC), choriocarcinoma (CH), yolk sac tumor (YST), and teratoma (TE) [1,2,3,4,5,6,7]. Overall, TGCTs represent a model of curable disease, with most patients presenting with stage I disease (around 70%), but approximately 75% of these are cured with orchiectomy alone, without the need for subsequent adjuvant treatments [4,8,9]. For those who require systemic therapy, TGCTs present extreme sensitivity to cisplatin-based chemotherapy, due to their unique molecular background [3,10]. However, a significant group of patients relapse, most frequently within the first two years after initial diagnosis [9,11]. Thus, inguinal orchiectomy followed by close surveillance may not be enough, implying the need for further treatments, which leads to higher morbidity [9].

Hence, this creates a clinical dilemma, which is to determine which patients actually do benefit from cytotoxic chemotherapy in the adjuvant setting to avoid disease relapse, discriminating them from those who can be safely put on surveillance. This is of extreme importance because these are very young patients who most likely will have to endure short and long-term side effects from chemotherapy during their lifetime [8,12,13,14,15]. Because of this, it is of paramount importance to identify reliable prognostic biomarkers that can predict patients who will most likely relapse, aiding in risk-stratification, particularly of stage I disease, and allowing the better tailoring of subsequent clinical management [8,13,16,17].

There have been attempts to discover further prognostic biomarkers, using widely available and reproducible techniques such as immunohistochemistry (IHC), available in all pathology departments [18]. Some of these biomarkers include Ki67, a marker of cell proliferation. In TGCTs, studies demonstrated that a poorer prognosis was associated with higher Ki67 expression; however, the cutoffs used varied, and results could not be validated in some studies and integrated in the clinic [9,14,19,20,21]. Other methodological differences contributed to controverse results [9,14,19,20,21]. Thus, the respective prognostic findings need validation in larger cohorts with standardized methodologies [9].

Another concern that is also related to biomarkers is finding those that can predict response to more targeted (and possibly less toxic) therapies. The epigenetic landscape of TGCTs and its influence also on tumor aggressiveness and response/resistance to cisplatin treatment make epidrugs promising agents [4,13,22,23,24,25]. In the result of epigenetic targeted therapy studies in TGCTs, other possible biomarkers have been identified, such as LSD1 [25,26,27]. LSD1 has been shown to be significantly elevated in pluripotent germ cell tumors, including TE, EC, and SE [28]. High levels of LSD1 are associated with overexpression of pluripotency genes, involved in cell growth and proliferation. When subjected to targeted therapy for LSD1, TGCT cell lines developed downregulation of those genes and growth arrest. Such finding can indicate a target for non-platinum-based therapies, making LSD1 a possible predictive epigenetic biomarker [29].

In this study, we aimed to determine the prognostic value of Ki67 and LSD1 in a well-defined cohort of TGCT patients, with the goal of finding a correlation with their outcomes. In addition, we attempted to verify a correlation between both markers related to tumor proliferation, and to assess the overall expression of LSD1 in TGCT subtypes, because this is a potential target for non-platinum targeted therapies.

## 2. Materials and Methods

### 2.1. Patients and Samples

In this retrospective study, a cohort of 157 TGCT patients (previously validated in [30]), diagnosed and treated by the same multidisciplinary team at the Portuguese Oncology Institute of Porto—Portugal (IPO Porto) between 2005 and 2019, was assessed. Clinical and histological data was reviewed according to the guidelines on the American Joint Committee on Cancer (AJCC) staging manual—8th edition, and the 2016 World Health Organization (WHO) Classification of Tumour of the Urinary System and Male Genital Organs. Patients with metastatic disease were further categorized according to the International Germ Cell Cancer Collaborative Group (IGCCCG) prognostic system. Follow-up was last updated in January 2021. Formalin-fixed and paraffin-embedded TGCT samples were selected, of which representative blocks (containing at least 1 cm^2^ of tumor) were thoroughly reviewed by a Pathologist experienced in TGCT evaluation. Four-micrometer-thick slides were ordered for immunostaining. Consultation cases, type I or III TGCT case, and cases with no adequate representative blocks available were excluded. Each sample was assessed globally for protein immunoexpression. In the presence of mixed tumors, each individual subtype was analyzed separately, resulting in a total of 221 individual TGCT histological samples separately analyzed.

This study is within the scope of a larger research project approved by the Ethics Committee of IPO Porto (Comissão de Ética para a Saúde, CES-IPO-1-018).

### 2.2. Immunohistochemistry

Ki67 immunohistochemistry was performed in an automated fashion using the Ventana platform as performed routinely for diagnostic purposes in the Department of Pathology, subjected to validation and quality control procedures. MIB-1 clone (DAKO) at 1:150 dilution was incubated for 1 h, and antigenic recovery was performed with cc1 for 36 min. The tissue of a normal tonsil was used as positive control.

In the case of LSD1, antigenic recovery was performed with EDTA buffer in a 30-min water-bath, and endogenous peroxidase activity was blocked by hydrogen peroxide in 3% methanol. Nonspecific reactions were blocked with normal horse serum (dilution 1:50). Slides were incubated for one hour at room temperature with primary LSD1 monoclonal antibody (clone #2139, Cell Signaling; dilution 1:225). Both post-primary antibody and polymer were incubated for 30 min at room temperature (Novolink^TM^ Polymer Detection System–Novocastra, Product No. RE7150-K, Leica Biosystems, Newcastle, UK). Diaminobenzidine was used as chromogen and hematoxylin for nuclear counterstaining. Tissue of normal prostate was used as positive control.

### 2.3. Digital Pathology Analysis

**Figure 1:**
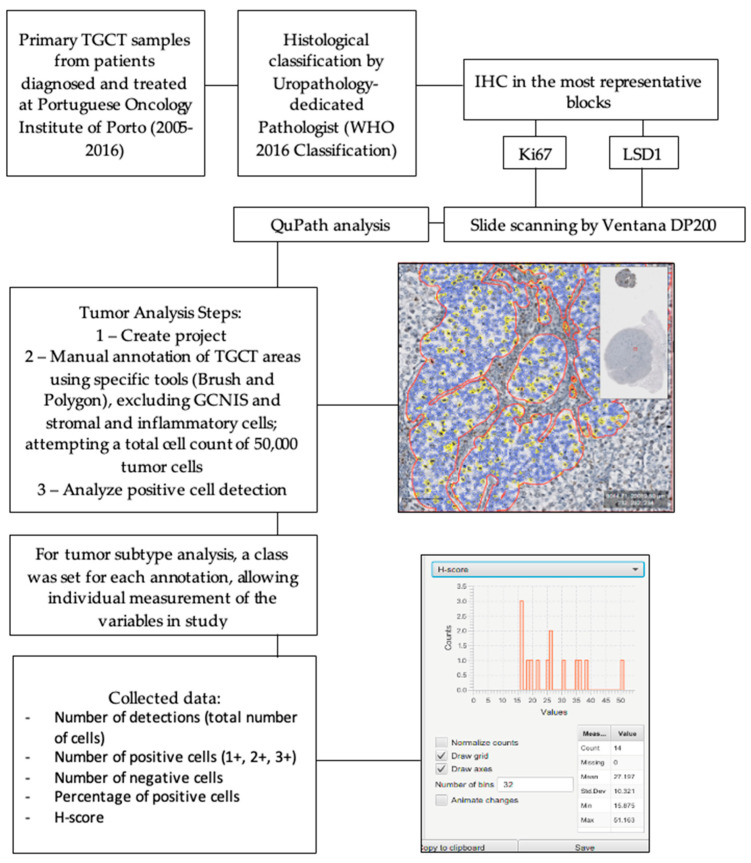
Study protocol. IHC, immunohistochemistry; TGCT, Testicular Germ Cell Tumor; WHO, World Health Organization.

Importantly, to improve the robustness of our results compared to previous studies, the immunoexpression of Ki67 and LSD1 was assessed using a digital image analysis system (QuPath, Version 0.2.3, QuPath developers, The University of Edinburgh, Scotland) for nuclear immunostaining quantification, after proper scanning, performed by VENTANA DP200 system. The detailed QuPath assessment and study protocol is represented in Figure 1. Briefly, QuPath integrated tools were used for area selection within the tumor, excluding GCNIS, stromal, and inflammatory cells. The manual selection tools allowed for clear “cell-by-cell” separation of highly intricated tumor components such as EC and YST, and accurate elimination of non-tumor cells. A total cell count of 50,000 tumor cells was attempted for every tumor sample. Within each selected area, positive cell analysis was conducted. Briefly, after setting nuclear, cell, and analysis parameters, including positive intensity threshold, the software automatically attributed expression score annotations to every cell (blue for no expression, yellow for low expression (+1), orange for moderate (+2), and red for high expression (+3)). From the annotation analysis results, the number of detected cells, number of positive detected cells (+1, +2, and +3), number of negative cells, positive cell percentage, and H-score (product of positive nuclei per each intensity score) were collected.

Importantly, for mixed tumors, the analysis included two contexts: first, a case-basis analysis, where randomly selected areas of the whole tumor area with representation of all components present in the slide were read; and second a component-basis analysis, where labels were attached to each tumor component in the slide (SE, EC, YST, CH, or TE), and analysis was carried out separately, creating multiple analysis metrics (as much as the number of components present in the slide).

### 2.4. In Silico Analysis

To corroborate our findings in an independent cohort, we explored The Cancer Genome Atlas (TCGA) database using the publicly available cBioPortal tool [31]. The PanCancer Atlas database was surveyed and mRNA expression values, according to tumor histological subtype and stage, were imported for MIB1 and LSD1 in the cohort of 149 patients.

### 2.5. Cell Lines and Treatments

The (T)GCT cell lines TCam-2 (representative of SE), NCCIT, 2102Ep, and NT2 (representative of NS) were cultured as described [32]. Cell lines have been previously authenticated (details reported in [33]).

NCCIT and NT2 cells were treated with differentiating agent all-trans retinoic acid—ATRA (STEMCELL^TM^ Technologies, Vancouver, BC, Canada) to generate differentiated cell lines from the parental ones, as described in [34]. Briefly, cells were seeded on T25 culture flasks, left to adhere for 24 h, and then treated with 10 µM of the drug for 10 days, with drug renewal every two days. Morphological evidence of differentiation has been confirmed (for details, see [33,34]).

### 2.6. Western Blot

Total protein was extracted from cells using the radioimmunoprecipitation assay buffer (Santa Cruz Biotechnology Inc., Dallas, TX, USA) complemented with 10% protein inhibitor cocktail. After 15 min on ice, samples were centrifuged at 13,000 rpm for 30 min at 4 °C, and the supernatant was collected. Protein was quantified using a Pierce BCA Protein Assay Kit (Thermo Scientific Inc., Waltham, MA, USA), according to manufacturer’s instructions. Aliquots of 30 µg total protein from each cell line were resuspended in loading buffer, denatured at 95 °C for 5 min, and loaded in 8% polyacrylamide gels, where they were separated by size through sodium dodecyl sulphate-polyacrylamide gel electrophoresis at 120 V. Then, proteins were transferred (semi-dry) to 0.2 μm polyvinylidene fluoride membranes (Bio-Rad Laboratories Inc., Hercules, CA, USA) using 25 mM Tris-base/glycine buffer and a Trans-Blot Turbo Transfer system (Bio-Rad) at 25 V and 1.3 mA for 20 min. Membranes were blocked with 5% bovine serum albumin (BSA; Santa Cruz, USA) and then incubated with the primary antibody mentioned above for LSD1, at 1:1000 dilution overnight at 4 °C, and with anti-ACTB (Sigma-Aldrich, A1978, 1/10,000 overnight at 4 °C). Lastly, membranes were incubated with secondary antibody coupled with horseradish peroxidase (Bio-Rad, Hercules, CA, USA), for 1 h at room temperature, at 1/5000 dilution (anti-mouse for ACTB and anti-rabbit for LSD1). Detection was performed by chemiluminescence. Quantification was performed using band densitometry analysis from the ImageJ software (version 1.6.1, National Institutes of Health, LOCI, Madison, WI, USA), by comparing the specific protein band intensity with the loading control beta-actin. Original blots are provided as supplementary material. Three biological replicates were used (two for ATRA-treated NT2 setting). ACTB gave a band on 48 KDa and LSD1 on 110 KDa, corresponding to their described molecular weight.

### 2.7. Statistical Analysis

Data was tabulated using Microsoft Excel for Office 365, and statistical analysis was performed using GraphPad Prism for Mac (version 9.1.1, GraphPad Software, San Diego, CA, USA) and SPSS Statistics for Mac (version 27.0.1.0, SPSS Inc, Chicago, IL, USA). Distribution of continuous variables between groups was compared using nonparametric tests, such as Mann–Whitney and Kruskal–Wallis tests. H-score correlation between Ki67 and LSD1 was assessed using Spearman test. Relapse/progression-free survival analysis was tested considering the 50th and 75th percentiles (P < 50, P ≥ 50, P < 75, and P ≥ 75). Survival curves were computed using the Kaplan–Meier method and log-rank test. Associations between categorical variables were assessed through Chi-square test. Statistical significance was set as *p* < 0.05. Statistical significance was annotated as such: * *p* < 0.05; ** *p* < 0.01; *** *p* < 0.001.

## 3. Results

### 3.1. Cohort Characterization

In total, 221 primary tumor components were studied, corresponding to a cohort of 157 patients. Median age at diagnosis was 31 years (interquartile range—IQR: 25–36) and median follow-up time was 69.0 months (95% CI 57.3–80.7); 52.2% of TGCT patients had pure SE, followed by 38.9% having mixed tumors. A summary of all demographic and clinicopathological data, including the proportion of individual tumor components, vascular invasion, tumor size, stage of disease, and IGCCCG prognostic group classification, is presented in Table 1.

### 3.2. Immunoexpression of Ki67 and LSD1

**Figure 2:**
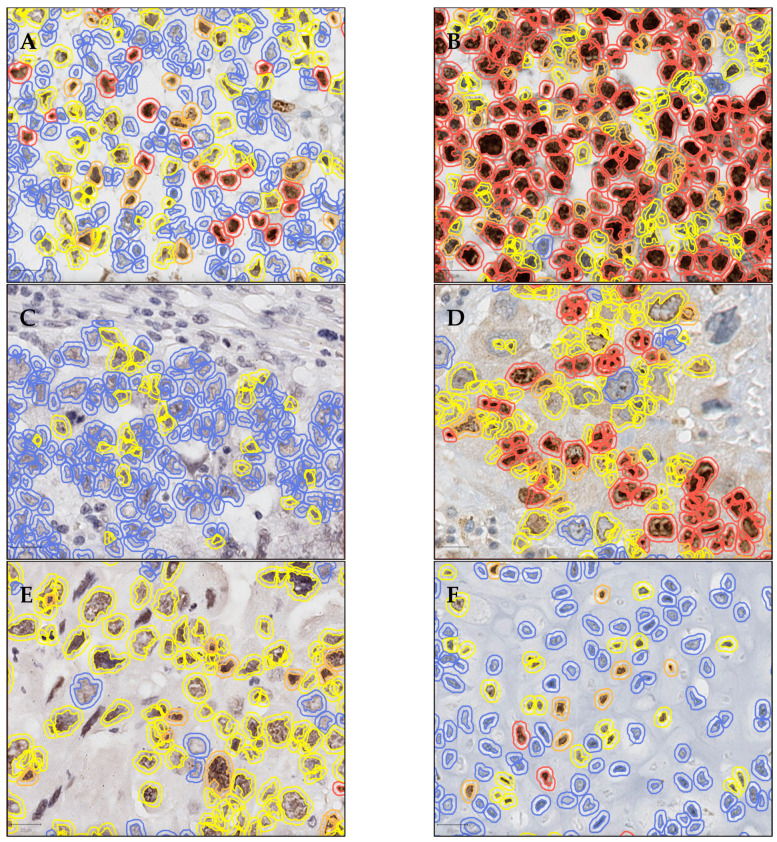
Illustrative examples of Ki67 and LSD1 immunoexpression across different testicular germ cell tumor subtypes, obtained through QuPath software with 40× magnification: (**A**). Case of pure SE, with Ki67 immunostaining; (**B**,**C**). Two cases of pure EC, with Ki67 and LSD1 immunostaining, respectively. Notice the several immune cells and stromal cells that were eliminated from the quantification; (**D**,**E**). CH components from the same mixed tumor case, with Ki67 and LSD1 immunostaining, respectively. Some cells are left uncounted for better appreciation of the nuclear DAB staining. (**F**). TE component of a mixed tumor case, with Ki67. The colors represent the intensity of expression (blue—no expression, yellow—low, orange—moderate, and red—high expression). Scale bars are presented for each photograph in the lower left corner.

Illustrative examples of Ki67 and LSD1 immunoexpression and analysis are depicted in Figure 2.

**Figure 3:**
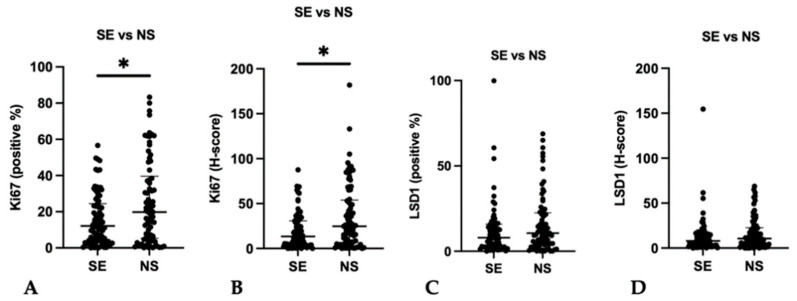
Immunoexpression in Seminomas and Non-seminomas. (**A**,**B**). SE versus NS; **A**. Ki67 positive %; (**B**). Ki67 H-score. (**C**,**D**). SE versus NS; **C**. LSD1 positive %; (**D**). LSD1 H-score. Median and interquartile range are presented. * *p* < 0.05. NS, Non-seminoma; SE, Seminoma.

An average 83,040 (range: 2733–47,0056) and 76,861 (range: 2231–49,6563) cells per sample were evaluated for Ki67 and LSD1, respectively. Median percentage of positive nuclei for Ki67 was 15.2% (IQR: 3.87–31.6), with a median H-score of 16.8 (IQR: 4.45–37.8). Median percentage of positive nuclei for LSD1 was 9.19% (IQR: 2.98–16.0), with a median H-score of 9.27 (IQR: 3.22–16.3). There was a significant positive (although weak) correlation between Ki67 and LSD1 H-scores (r_s_ = 0.182, *p* = 0.037). Expression of Ki67 was significantly higher in NS compared to SE (Table S1, Figure 3), both considering percentage of positive nuclei (*p* = 0.0316) and H-score (*p* = 0.0113). Although LSD1 expression was overall higher in NS, it did not achieve statistical significance (*p* = 0.1973 and *p* = 0.1630 for percentage of positive nuclei and for H-score, respectively) (Table S1 and Figure 3). Similar results occurred for the expression between pure forms (SE, EC, and TE) and mixed tumors, with no significant differences observed (*p* = 0.1404 and *p* = 0.1677 for percentage of Ki67 positive nuclei and for H-score, and *p* = 0.9218 and *p* = 0.8658 for LSD1 percentage of positive nuclei and for H-score, respectively, Figure S1).

**Figure 4:**
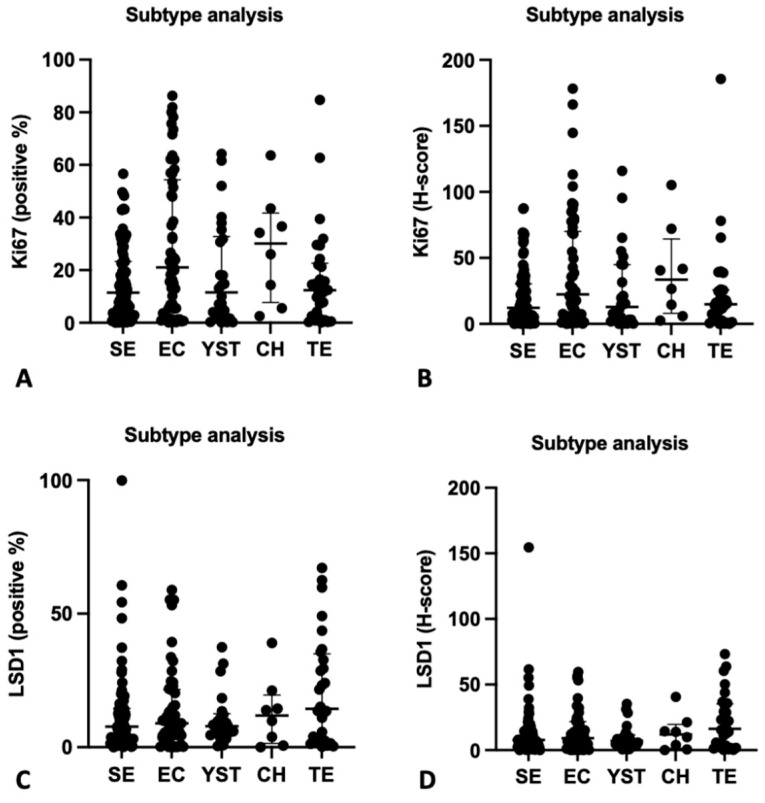
Immunoexpression among TGCT histological subtypes (SE, EC, YST, CH, and TE). (**A**). Ki67 positive %; (**B**). Ki67 H-score; (**C**). LSD1 positive %. (**D**). LSD1 H-score. Median and interquartile range are presented. CH, Choriocarcinoma; EC, Embryonal Carcinoma; NS, Non-seminoma; SE, Seminoma; TE, Teratoma; YST, Yolk-sac tumor.

When considering individual tumor subtypes, expression of Ki67 was overall higher in CH, followed by EC; however, differences towards other components did not achieve significance after correcting for multiple comparisons (*p* = 0.0548 and *p* = 0.1292 for percentage of positive nuclei and H-score, respectively) (Table S1, Figure 4). Similarly, for LSD1, expression was higher in TE, CH, and EC, but differences did not achieve significance upon correcting for multiple comparisons (*p* = 0.3275 and *p* = 0.2824 for percentage of positive nuclei and H-score, respectively) (Table S1 and Figure 4).

### 3.3. Association with Clinicopathological Features

The expression of both Ki67 and LSD1 was not significantly associated with clinicopathological features, including stage of disease, the IGCCCG prognosis-based system, and vascular invasion.

The same analysis for stage was computed individually only for SE and only for NS subgroups of patients, again depicting no statistically significant differences (Table 2).

Regarding disease relapse/progression, expression of both Ki67 and LSD1 did not significantly associate with the event of relapse and there were no significant differences regarding relapse/progression-free survival (Figure S2).

### 3.4. In Silico Study—cBioportal Data

Corroborating the IHC findings, in silico analysis of the TCGA database showed no significant difference in the expression of these markers between different histological subtypes or according to AJCC disease stage (Table 3).

### 3.5. In Vitro Study—LSD1 Expression in TGCT Cell Lines

**Figure 5:**
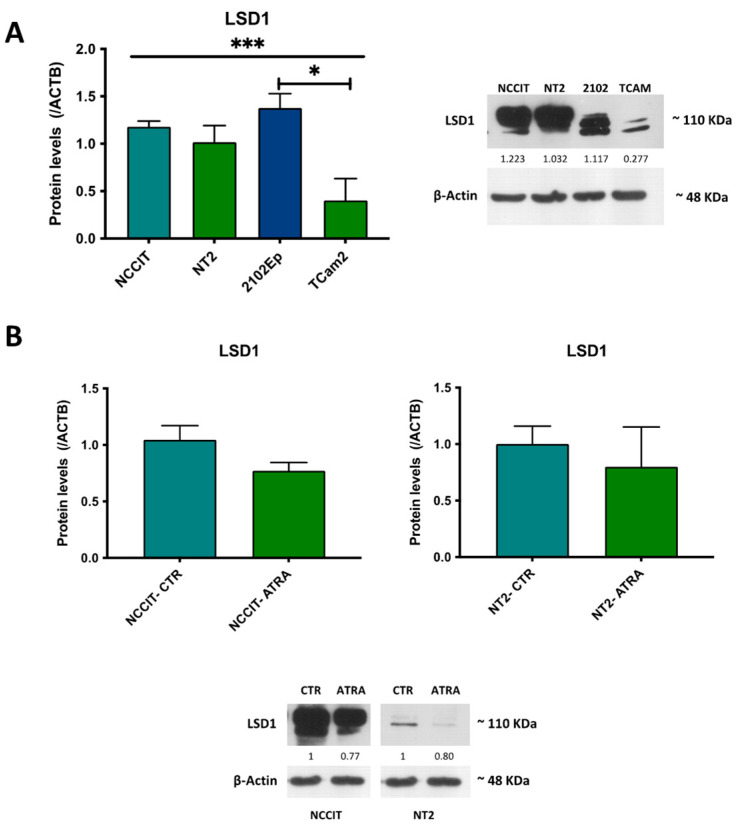
LSD1 protein expression in TGCT cell lines. (**A**)—All cell lines; (**B**)—NCCIT versus differentiated NCCT (ATRA-treated) and NT2 versus differentiated NT2 (ATRA-treated). Results are normalized to ACTB (and to vehicle/control in (**B**)). Abbreviations: CTR—control. Representative bands and densitometric readings are presented as assessed by ImageJ software. Non-parametric tests were used (Kruskal–Wallis and Mann–Whitney U-test, as appropriate). * *p* < 0.05, *** *p* < 0.01.

LSD1 was differentially expressed among cell lines (*p* = 0.0006). LSD1 expression was higher in NCCIT, NT2, and 2102Ep cell lines, representative of NS (Figure 5A). TCam-2, representative of SE, showed the lowest expression, being significantly lower when compared to 2102Ep when adjusting to multiple comparisons (adjusted *p*-value of 0.0134).

Additionally, LSD1 protein expression decreased in differentiated (ATRA-treated) NCCIT, and NT2 cell lines derived from the original clone (Figure 5B).

## 4. Discussion

In this study, we used a digital imaging analysis system for IHC analysis in TGCTs, increasing the robustness of data acquired regarding the clinical impact of such biomarkers compared to previous investigations. QuPath is a new platform created for image analysis, with an increasing interest found in oncology research. Digital pathology is revolutionizing pathology practice and research, fulfilling the need for accurate biomarker analysis in leading reference hospitals; the need for a cost-effective tool with reproducible, consistent, and accurate results that is universally applicable; and the requirement for solutions allowing remote pathology diagnosis, for instance, in the context of pandemics [35].

Initial studies indicated that Ki67, originally identified in the 1980s, is detected in dividing cells (G1, S, G2, and M phase) and not in quiescent cells (G0 phase), with higher expression levels early in mitosis [36,37,38]. Despite a poor understanding of the extent of the functions and dynamics of Ki67, this protein is widely used as a proliferation index, which is assessed through IHC in tumor sections [39]. Many authors propose that the proliferative activity appraisal in several malignant tumors correlates with aggressiveness, progression, and metastatic behavior [40]. Consequently, Ki67 positive nuclei scores may predict prognosis, as well as the potential response to treatment and its benefit [39,41]. The reliability of this marker has been shown in several types of cancer [39,42]. It is actually part of the routine diagnostic assessment of breast cancer and neuroendocrine tumors [38,39].

Tumor proliferative activity has been correlated with occult metastatic disease in patients with low stage NS [40,43]. However, in spite of statistically significant differences found between NS patients with or without metastatic disease, Ki67 staining still has not shown clinical utility to predict patients at high risk for metastasis [37]. This had been explained by the lack of precise discrimination between risk groups with highly proliferating tumors, such as NS, because Ki67 recognizes all cells that have entered the cell cycle [37]. Our study corroborated such findings, showing no significant association between Ki67 positive nuclei percentage or H-score and disease stage (Table 2). Our mean Ki67 positive nuclei percentage was 15.2% (19.8% for NS), which, according to previous studies, are lower values [37]. This may be due to the antibody clone used and to the more precise digital methodology employed, with elimination of staining from infiltrating immune cells and stromal cells, which contribute substantially to the overall staining observed in tumor sections. Furthermore, our data was validated by the in silico analysis of cBioPortal, also showing no differences in expression according to stage (Table 3).

Although we showed higher Ki67 expression in NS compared to SE, in line with the higher proliferative rates of the more aggressive NS tumors, this was not observed in the analysis of TCGA data, which may be due to assessment of mRNA data only (compared to protein immunoexpression in our study) and to differences in cohort composition. Interestingly, higher expression levels were overall seen in highly aggressive tumor components, CH and EC, which are frequently of poor prognosis. This may reflect some inner aggressiveness of these tumors related to their biology, but our results of Ki67 staining distribution among the several subtypes show that TGCTs are indeed highly heterogeneous, and a wide proportion of proliferating cells may be found in these tumors.

Ki67 scoring is overall a controversial topic in several cancers, partly because of the considerably heterogenous cutoffs, study, laboratorial, and analytical methods [38,39,44]. Given the non-uniform IHC protocols among distinct laboratories, as well as many detection systems and interpretations methods, the results among numerous articles in the same fields may differ in a way that precludes proper and definitive conclusions [45,46]. Considering the methodology of studies in TGCTs, the main differences start with the IHC, conducted in some by standard manual techniques [20,21,37,40,47], while others applied automated platforms [9,14]. The type of sample used for assessment is also a significant factor that may change the results. While some articles used whole tissue slides of a representative tissue block [20,21,37,40,47], others used tissue microarrays (TMA) [19], and others counted expression only in the EC components of NS tumors [21]. Although more practical for screening purposes, TMAs may underestimate the proper assessment of a tumor sample, not representing adequately the tumors, especially when focusing on heterogenous cancers such as TGCT [48,49]. Specific subtypes of mixed tumors may possibly be under-represented, implicating a variety of observations, with consequent different interpretations of clinicopathological features, namely the prognosis [49], especially knowing that mixed tumor components may be biologically different from the pure corresponding tumors [50]. With our case-basis approach, representing random areas of all histological components present in mixed tumors, we aimed to represent an average expression of both biomarkers in tissue samples (instead of preferentially selecting EC components as has been done in the past) [21]; at the same time, the component-basis analysis allowed for more biological-driven interpretations, related to the properties of each histological element.

Cutoffs used for Ki67 expression in TGCT works are also a contributing factor for the diverseness of results, particularly regarding prognostic evaluation. The most studied cutoff is 70% [9,14,20,21]. Other cutoffs were also considered, such as 40% and 80% [9,14,40]. However, as further studies could not prove such correlation with the given cutoffs, others were proposed, such as 50% [9]. Nonetheless, the association with prognosis was not always verified, particularly when adjusting to other clinicopathological features such as vascular invasion [9,14]. While Ki67 seemed to be, initially, an interesting prognostic biomarker when subjectively assessed through rough “eyeball” quantification, a myriad of different results was rapidly obtained, and its significance was lost, particularly when adjusted for other variables. Indeed, several studies (summarized in Table S2, which presents a literature review on this topic) show that Ki67 either fails to have important clinicopathological correlates or loses its prognostic impact when adjusting to other variables in multivariable models. Additionally, Ki67 was shown not to be useful for truly identifying a high-risk group, because half of the patients with Ki67 > 70% showed no metastatic disease [21]. Our digital pathology and more objective analysis further confirm that Ki67 is not a promising prognostic biomarker to be introduced in the clinical setting for guiding treatment decisions for TGCT patients.

Epigenetic chromatin modifications are widely known to play an important role in tumorigenesis [26,27]. Lysine-specific demethylase 1 (LSD1), also known as KDM1A, is one of the many epigenetic markers currently under study [26,27]. LSD1 is the first identified histone lysine demethylase responsible for histone demethylation, specifically of monomethyl and dimethyl histone 3 at lysine 4 (H3K4) and 9 (H3K9), where H3K4 marks an active chromatin transcription state and H3K9 a repressive one [26]. The consequence of histone demethylation, depending on the location and degree of residue methylation, is the regulation and control of transcription, differentiation, stemness, cell motility, epithelial-to-mesenchymal transition, metabolism, autophagy, and senescence [26,51,52]. Indeed, high levels of LSD1 are associated with the expression of pluripotent stem cell markers, such as OCT4 and SOX2, as well as with repression of p53 and long noncoding RNA (lncRNAs) complexes, with consequent sustaining of cell stemness and upregulation of oncogenic pathways [26,27,51,53].

LSD1 has been reported to be overexpressed in a variety of cancers, such as lung, bladder, prostate, brain, colorectal, and breast cancers and hematologic malignancies, determining poor prognosis [25,51]. In studies with pluripotent germ cell tumors, LSD1 exhibited significant expression levels, namely in teratocarcinoma, EC, and SE cells, in contrast with normal testicular tissue [28,54]. In our study, we also confirmed that TGCT cell lines express LSD1, and showed that NS-related cell lines with predominant EC component show the highest expression levels (including NCCIT, representative of an aggressive primary mediastinal EC, Figure 5A). Furthermore, we also show that expression is slightly reduced upon differentiation of NCCIT and NT2 cells with ATRA (Figure 5B), which is in line with the described function of LSD1 related to stemness [26,27,51,53]. This in vitro investigation also supported the specificity of the antibody used and led us to explore it at the tissue level.

This way, we also analyzed comprehensively LSD1 immunoexpession in a large and well-characterized TGCT tissue cohort, which has not been done before. In our study, however, we found no significant differences in expression of LSD1 among the various histological subtypes, and this was confirmed in TCGA cohort analysis. Higher levels were seen overall in very distinct subtypes, such as TE and more undifferentiated subtypes such as EC (Figure 4). This may point to a broad function of LSD1 in these tumors in actual patients.

There was also no significant correlation between LSD1 positive nuclei percentage or H-score and disease stage, nor with relapse/progression-free survival, limiting its use as a prognostic marker. However, finding LSD1 expression in all histological subtypes of TGCTs in such a large cohort still may lead to exploring LSD1 inhibitors as therapeutic targets for all TGCT patients, regardless of histology. Indeed, using LSD1 inhibitors with the purpose of exploring the biological function of this enzyme, Wang et al. found that such inhibition selectively blocked the growth of TE, EC, SE, and embryonic stem cells, also seen in other recent studies [28,53]. Due to the potential of LSD1 as a target for cancer therapy, there are several LSD1 inhibitors being explored, in which the overall observed effects consisted of the inhibition of cell growth, proliferation, and mobility, and also regulation of transcription, including up- and downregulation of particular genes [52]. Although promising as a target therapy, with significant results in clinical trials for small cell lung cancer, glioma, prostate, and breast cancer, issues related to safety and pharmacodynamics still need to be overcome [52]. More studies are needed to uncover the usefulness of LSD1 inhibitors in TGCT patients.

Notably, we found a significant correlation between the expression of Ki67 and LSD1 H-score. Although this correlation was week, it seems to corroborate that tumors with higher proliferation are those with higher LSD1 expression, which relates to stemness properties.

To conclude, despite individual studies that may have suggested significant utility in evaluating Ki67 through the pathologist’s eye by a non-digital quantification, our study shows that such utility in a randomly selected cohort, from daily practice, cannot be demonstrated. In silico analyses corroborate our results regarding both biomarkers and the lack of association with outcome and other clinical features. LSD1, while limited as a prognostic biomarker, may be a potential therapeutic target expressed overall in various histological subtypes. It requires more well-defined studies, given the promising role of such treatments.

## Figures and Tables

**Table 1:** Demographic and clinicopathological characteristics of the study cohort.

Variables	Patient Cohort (*n* = 157) Tumor Samples (*n* = 221)
Age (years) (median, IQR)	31 (25–36)
Laterality (*n*, %)	
Right	83/153 (54.2)
Left	68/153 (44.5)
Bilateral synchronous	2/153 (1.3)
Pre-operative AFP (*n*, %)	
Within normal range	97/151 (64.2)
Elevated	54/151 (35.8)
Pre-operative *β*-HCG (*n*, %)	
Within normal range	73/151 (48.3)
Elevated	78/151 (51.7)
Pre-operative LDH (*n*, %)	
Within normal range	71/124 (57.3)
Elevated	53/124 (42.7)
Histologic subtypes (*n*, %)	
Pure SE	82/157 (52.2)
Pure EC	11/157 (7.0)
Pure TE	3/157 (1.9)
Mixed Tumor	61/157 (38.9)
Tumor components (*n*, %)	
SE	96/221 (43.5)
EC	56/221 (25.3)
CH	10/221 (4.5)
YST	27/221 (12.2)
TE	32/221 (14.5)
Stage AJCC8 (*n*, %)	
I	100/156 (64.1)
II	32/156 (20.5)
III	24/156 (15.4)
Vascular Invasion (*n*, %)	
Yes	78/157 (49.7)
No	79/157 (50.3)
Tumor largest dimension (cm) (median, range)	4.7 (0.7–18)
Metastatic disease at diagnosis (*n*, %)	
Yes	56/156 (35.9)
No	100/156 (64.1)
IGCCCG prognosis group (with metastatic disease) (*n*, %)	
Good	42/56 (75)
Intermediate	8/56 (14.3)
Poor	6/56 (10.7)
Relapse/Progression (*n*, %)	
Yes	10/157 (6.4)
No	147/157 (93.6)
Additional treatments (*n*)	
RT	46
CT	99
Vital status (*n*, %)	
Alive with no disease	151/157 (96.2)
AWD	3/157 (1.9)
DFD	2/157 (1.3)
D-NED	1/157 (0.6)

AJCC, American Joint Committee on Cancer; AFP, Alpha-fetoprotein; AWD, Alive with disease; CH, Choriocarcinoma; CT, Chemotherapy; DFD, Died from disease; D-NED, Died with no evidence of disease; EC, Embryonal Carcinoma; IGCCCG, International Germ Cell Cancer Collaborative Group; IQR, Interquartile Range; LDH, Lactate dehydrogenase; RT, Radiotherapy; SE, Seminoma; TE, Teratoma; YST, Yolk-sac Tumor.

**Table 2:** Immunoexpression of Ki67 and LSD1 according to clinicopathological features.

Stage	I	II		III	*p*-Value
**Ki67**					
Positivity (%) (median (IQR))	15.4 (4.7–32.2)	10.8 (3.4–24.0)		21.7 (4.3–30.1)	0.593
Only SE	13.9 (3.87–25.4)	6.6 (3.19–17.6)		11.1 (5.17–31.2)	0.791
Only NS	22.8 (5.9–58.5)	14.1 (3.4–32.0)		22.1 (3.6–30.5)	0.278
H-score (median (IQR))	17.6 (5.0–42.1)	11.2 (3.9–26.6)		24.6 (5.1–35.9)	0.490
Only SE	14.5 (4.2–31.5)	6.8 (3.5–19.9)		12.3 (6.4–31.9)	0.715
Only NS	25.5 (6.3–76.2)	14.2 (4.4–35.8)		24.8 (4.4–38.6)	0.274
**LSD1**					
Positivity (%) (median (IQR))	8.2 (2.8–17.2)	9.4 (2.3–13.7)		10.6 (3.2–15.6)	0.894
Only SE	7.8 (2.6–15.8)	9.8 (2.5–15.7)		13.4 (3.1–16.2)	0.953
Only NS	9.8 (3.7–21.6)	9.0 (1.6–13.7)		9.2 (3.2–15.1)	0.692
H-score (median (IQR))	8.2 (3.1–17.3)	9.4 (2.1–13.9)		10.6 (3.2–15.6)	0.879
Only SE	7.9 (2.6–15.8)	9.8 (2.5–15.7)		13.4 (3.1–16.2)	0.960
Only NS	9.8 (4.3–22.2)	9.1 (1.6–14.0)		9.3 (3.2–15.3)	0.644
**IGCCCG**	**Good**	**Intermediate**		**Poor**	***p*-value**
**Ki67**					
Positivity (%) (median (IQR))	10.4 (3.2–26.1)	20.7 (4.9–41.5)		23.9 (17.6–28.4)	0.207
H-score (median (IQR))	11.9 (3.7–27.7)	23.3 (5.0–75.1)		29.3 (20.1–35.8)	0.179
**LSD1**					
Positivity (%) (median (IQR))	10.6 (1.6–15.6)	5.8 (4.2–40.9)		6.7 (3.3–13.5)	0.977
H-score (median (IQR))	10.7 (1.6–15.6)	5.8 (4.2–41.9)		6.7 (3.4–13.7)	0.979
	**Vascular Invasion**		**No Vascular Invasion**		***p*-value**
**Ki67**					
Positivity (%) (median (IQR))	17.6 (3.6–33.4)		13.9 (4.6–29.3)		0.364
H-score (median (IQR))	18.6 (4.3–41.7)		15.4 (4.9–36.3)		0.470
**LSD1**					
Positivity (%) (median (IQR))	9.0 (2.4–16.1)		9.4 (3.3–15.1)		0.738
H-score (median (IQR))	9.1 (2.4–16.2)		9.4 (3.6–16.5)		0.684

IGCCCG, International Germ Cell Cancer Collaborative Group; IQR, Interquartile Range; NS, Nonseminoma; SE, Seminoma.

**Table 3:** In silico analysis of TCGA data.

SE vs. NS				
	SE	NS		*p*-Value
Ki67 mRNA expression (median (IQR))	888 (635–1088)	758 (505–1122)		0.262
LSD1 mRNA expression (median (IQR))	4012 (3220–4426)	3903 (2735–5650)		0.311
Stage				
	I	II	III	*p*-value
Ki67 mRNA expression (median (IQR))	806 (635–1115)	592 (424–1054)	856 (424–1133)	0.216
LSD1 mRNA expression (median (IQR))	3956 (3214–5018)	4216 (2636–5340)	4762 (3208–5841)	0.437

AJCC, American Joint Committee on Cancer; IQR, Interquartile Range; mRNA, messenger RNA; NS, Nonseminoma; SE, Seminoma.

## Data Availability

Not applicable.

## Supplementary Materials

The following are available online at https://www.mdpi.com/article/10.3390/life12020264/s1, **Figure S1**. Immunoexpression in pure TGCT subtypes (SE, EC, and TE) versus Mixed type. A. Ki67 positive %; B. Ki67 H-score; C. LSD1 positive %. D. LSD1 H-score. Median and interquartile range are presented. EC, Embryonal Carcinoma; NS, Non-seminoma; SE, Seminoma; TE, Teratoma. **Figure S2**. Relapse/Progression free survival according to biomarkers 50th (P50) and 75th (P75) percentiles. A–B. Relapse/progression free survival according to P50. A. Ki67 H-score; B. LSD1 H-score; C–D. Relapse/progression free survival according to P75; C. Ki67 H-score; D. LSD1 H-score. **Table S1.** Expression of Ki67 and LSD1 according to histology. **Table S2.** Review of literature regarding TGCT and the expression of Ki67.
