# Supplementary material for: Ki67 and LSD1 Expression in Testicular Germ Cell Tumors Is Not Associated with Patient Outcome: Investigation Using a Digital Pathology Algorithm
Source: Life (Basel). 2022 Feb 10;12(2):264. doi: 10.3390/life12020264 (PMC8875543; doi:10.3390/life12020264)
Supplement: Supplementary file 1 [file life-12-00264-s001.zip › life-12-00264-s001/life-1596019-supplementary.pdf]

Article

Ki67 and LSD1 Expression in Testicular Germ Cell Tumors Is not Associated with Patient Outcome: Investigation Using A Digital Pathology Algorithm

Beatriz Chaves Lourenço [1], Catarina Guimarães-Teixeira [2], Bianca C. T. Flores [2], Vera Miranda-Gonçalves [2,3], Rita Guimarães [1], Mariana Cantante [1], Paula Lopes [1], Isaac Braga [4], Joaquina Maurício [5], Carmen Jerónimo [2,3], Rui Henrique [1,2,3,*,†] and João Lobo [1,2,3,*,†]

[1] Department of Pathology, Portuguese Oncology Institute of Porto (IPOP), R. Dr. António Bernardino de Almeida, 4200-072 Porto, Portugal; up201605750@icbas.up.pt(B.C.L.), rita.guimaraes@ipoporto.min-saude.pt(R.G.); mferreira@ipoporto.min-saude.pt(M.C.); ana.ambrosio@ipoporto.min-saude.pt(P.L.)

[2] Cancer Biology and Epigenetics Group, Research Center of IPO Porto (CI-IPOP)/RISE@CI-IPOP (Health Research Network), Portuguese Oncology Institute of Porto (IPO Porto)/Porto Comprehensive Cancer Center (Porto.CCC), R. Dr. António Bernardino de Almeida, 4200-072 Porto, Portugal; catarina.guimaraes.teixeira@ipoporto.min-saude.pt(C.G.-T.); bianca.troncarelli@ipoporto.min-saude.pt(B.C.T.F.); Vera.Miranda.Goncalves@ipoporto.min-saude.pt(V.M.-G.); carmenjeronimo@ipoporto.min-saude.pt(C.J.)

[3] Department of Pathology and Molecular Immunology, ICBAS – School of Medicine and Biomedical Sciences, University of Porto (ICBAS-UP), Rua Jorge Viterbo Ferreira 228, 4050-513 Porto, Portugal

[4] Department of Urology, Portuguese Oncology Institute of Porto (IPOP), R. Dr. António Bernardino de Almeida, 4200-072 Porto, Portugal; isaac.braga@ipoporto.min-saude.pt(I.B.)

[5] Department of Medical Oncology, Portuguese Oncology Institute of Porto (IPOP), R. Dr. António Bernardino de Almeida, 4200-072 Porto, Portugal; jmauricio@ipoporto.min-saude.pt(J.M.)

* Correspondence: henrique@ipoporto.min-saude.pt(R.H.); jpedro.lobo@ipoporto.min-saude.pt/joaomachadolobo@gmail.com(J.L.)

† joint senior authors.

Supplementary Figure S1. Immunoexpression in pure TGCT subtypes (SE, EC, and TE) versus Mixed type.

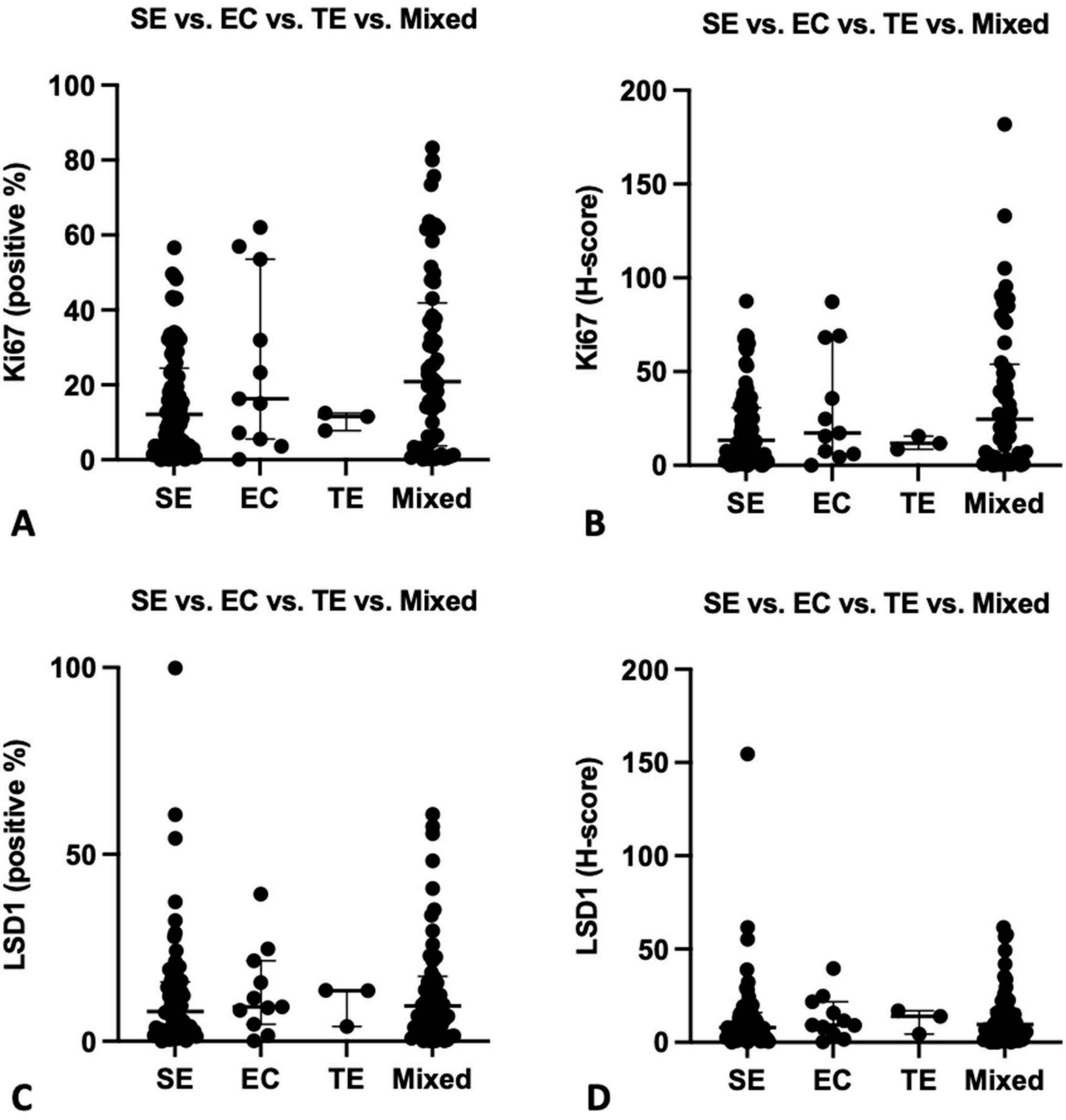

A. Ki67 positive %; **B**. Ki67 H-score; **C**. LSD1 positive %. **D**. LSD1 H-score. Median and interquartile range are presented. EC, Embryonal Carcinoma; NS, Non-seminoma; SE, Seminoma; TE, Teratoma.

Supplementary Figure S2. Relapse/Progression free survival according to biomarkers 50th (P50) and 75th (P75) percentiles.

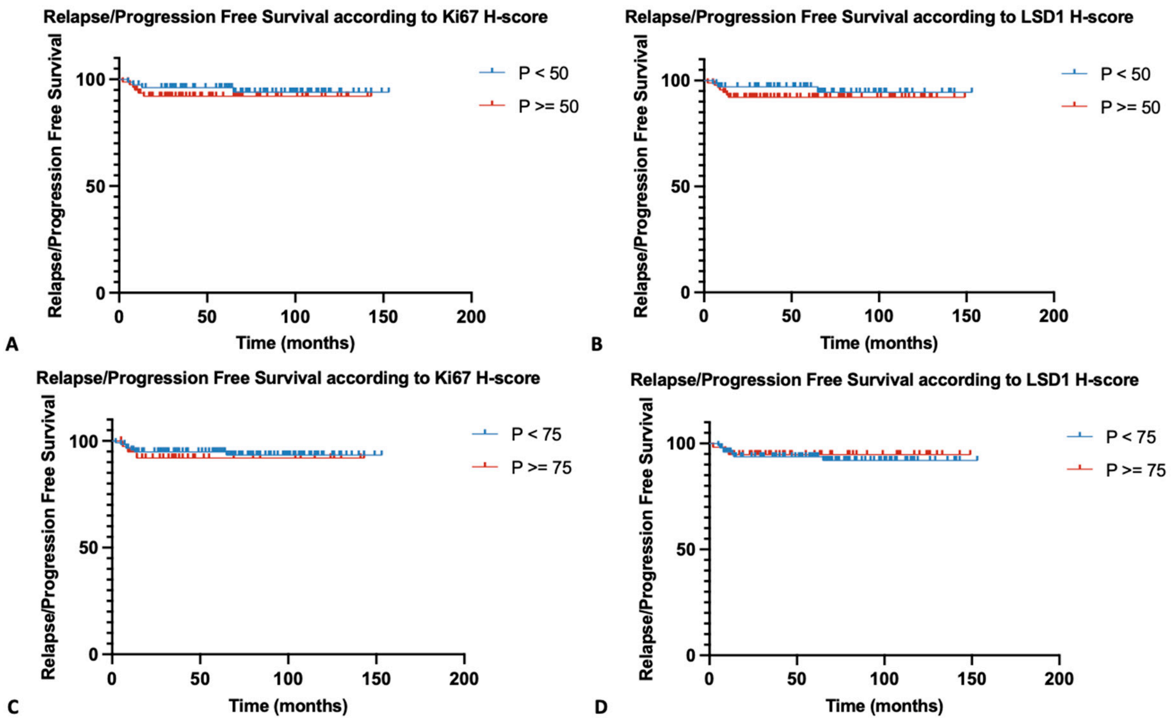

A–B. Relapse/progression free survival according to P50. **A.** Ki67 H-score; **B.** LSD1 H-score; C–D. Relapse/progression free survival according to P75; **C.** Ki67 H-score; **D.** LSD1 H-score.

Supplementary Table S1. Expression of Ki67 and LSD1 according to histology.

	SE	NS	*p*-value
**Ki67**			
Positivity (%) (median [IQR])	12.2 (3.7–24.5)	19.8 (5.3–39.6)	0.0316 *
H-score (median [IQR])	13.4 (4.0–87.5)	24.7 (6.7–53.9)	0.0113 *
**LSD1**			
Positivity (%) (median [IQR])	8.0 (2.6–15.9)	10.6 (4.2–22.6)	0.1973
H-score (median [IQR])	8.0 (2.6–15.9)	10.6 (4.3–22.6)	0.1630

	SE	EC	YST	CH	TE	*p*-value
**Ki67**						
Positivity (%) (median, [IQR])	11.5 (3.6–23.3)	21.0 (4.9–54.3)	11.6 (2.8–32.8)	30.1 (7.7–41.7)	12.4 (2.43–22.6)	0.0548
H-score (median, [IQR])	12.3 (4.0–30.4)	22.5 (5.4–70.2)	12.9 (2.9–44.9)	33.6 (8.1–64.4)	14.9 (2.5–25.7)	0.1292
**LSD1**						
Positivity (%) (median, [IQR])	7.8 (2.5–14.6)	9.0 (4.1–21.6)	7.9 (4.7–12.5)	11.9 (1.5–19.5)	14.4 (2.7–34.9)	0.3275
H-score (median, [IQR])	7.8 (2.5–14.6)	9.0 (4.1–21.8)	7.1 (4.3–11.6)	11.9 (1.5–19.5)	16.2 (2.7–35.2)	0.2824

CH Choriocarcinoma, EC Embryonal Carcinoma, IQR Interquartile Range, NS Nonseminoma, SE Seminoma, TE Teratoma, YST Yolk-sac tumor, * significant values.

Supplementary Table S2. Review of literature regarding TGCT and the expression of Ki67.

Reference	Cohort size, Sample type and Stage of Disease	Number of SE and NS (SE/NS)	Date of Diagnosis (interval)	IHC	Methodology	Outcome
Lobo et al. (2020) [9]	70 TGCT with Stage I	28/42	1993-2018 (median follow-up of 42 months)	Automated	Performed by a TGCT-dedicated investigator, grading from 0 to 3, using 40 and 70% cutoffs for Ki67	Although relapse free survival was better for patients with ≤70%, no significant association was found between Ki67 staining percentage and the event of relapse (p=0.127). Adjusting for the effect of vascular invasion, Ki67 staining loses its impact on relapse-free survival. Considering patients without vascular invasion, those with Ki67 > 50% experienced worse outcomes (p=0.042).
Gilbert et al. (2015) [14]	190 NS with stage I, with additional TMAs (59 and 80, managed with surveillance and minimum follow-up of 2 years, from different hospitals)	0/190	From TE08 trial: 1998-2003 (median follow-up of 40 months)	Automated	Performed by two independent pathologists, recording intensity of staining (from 0 to 3), and percentage of positive staining, using 40 and 70% cutoffs for Ki67	Ki67 expression was significantly associated with decreasing likelihood of the tumor containing SE, and increasing likelihood of vascular invasion. There was no additional prognostic value for Ki67 staining, for either one of the used cutoffs. Patients with weak staining had better prognosis, but such finding was associated with vascular invasion (independent value was not corroborated in multivariable analysis).
Gallegos et al. (2011) [17]	62 Pure Seminomas:<br>43 in stage I<br>17 in stage II<br>2 in stage III	68/0	1996-2005	Automated	Reviewed by 2 pathologists, using tissue microarrays (TMA)	There was no significant association between Ki67 and metastatic disease at diagnosis. There was a significant inverse association between *rete testis* invasion and Ki67 expression higher than 50%.
P. Albers et al. (2003) [18]	152 patients with NS, assigned for retroperitoneal lymph node resection (RPLNR)	0/152	1996-2002 (median follow-up of 34,5 months)	Performed on paraffin sections (5 µm) stained with MIB-1 antibody by standard technique.	Performed by one pathologist, who counted and calculated the percentage of Ki67 positively stained nuclei in more than 500 tumor cells (Ki67 score: Ki67 positive tumor cells/total number of tumor cells). A cutoff of 70% was used to test the accuracy of its predictive value. When possible, Ki67 score was assessed only in EC components.	Mean Ki67 score was 57.9 and 71.9 for Stage I and II, respectively. It was significantly higher for stage II (p=0.003). Using a cutoff of 70%, a negative predictive value of 78% and a positive predictive value of 47.3% were obtained. The combination of Ki67 score with vascular invasion was able to predict a low-risk group at the 86.5% level.

Albers et al. (1996) [19]	78 NS with clinical stage I (50 and 28 with pathologic Stage I and II, respectively)	0/78	1983-1994 (median follow-up 58.2 months)	Performed on paraffin sections stained with Ki67 antibody by standard technique	Performed by one pathologist, who manually counted Ki67 positively stained tumor cells at 1400 and calculating the percentage of positively stained nuclei of the total number of tumor cells. A cutoff of 70% was used. When possible, Ki67 score was assessed only in EC components.	The Ki67 mean value was significantly higher in stage II disease (p=0.02). A cutoff value of 70% Ki67 positive nuclei was best to predict the pathologic stages. The prediction of a low-risk group was possible with an accuracy of approximately 90%. However, Ki67 was not useful in predicting a high-risk group, because half of the patients with Ki67 values > 70% had no metastasis (positive predictive value of 55%).
Albers et. al (1995) [38]	62 Nonseminomatous TGCT (with 45 in Stage A and 17 with metastatic disease)	0/62	1992 (median follow-up of 19.9 months)	Performed on paraffin sections stained with MIB-1 antibody by standard technique, using a modification of the method of Cattoretti et al.	Performed by 2 pathologists experienced in this practice, who manually assessed Ki67 scoring of the positively stained nuclei, with a cutoff of 80%, performed in the most intensely stained well-fixed areas of the tumors with the exclusion of seminomatous, teratomatous and/or stromal components.	The mean values of Ki67 positive expression were 66.3% and 80.2% for stage A and metastatic-group patients, with a statistically significant difference (p=0.0032).
Mazumdar et al. (2003) [45]	95 NSGCT (all containing EC alone or in association with other NS components). Only EC components were used in this study.	0/95	1975-1996 (median follow-up of 9.3 years)	Performed on paraffin sections (4 μm), from paraffin-embedded archival tissue blocks) by standard technique using streptavidin-biotin antibodies against Ki67 (diluted to 1:100)	Not described	Hierarchical agglomerative cluster analysis was used with the intention of discovering a subgroup of patients, assessing Ki67, p53, apoptosis, AFP and β-HCG. Cluster A patients (37) show higher Ki67 values, of whom 70% and 60% had good and poor prognosis, respectively. The 5-year survival rate was 94%. Data suggested that Ki67, p53 and apoptosis signaling pathways would be relevant to clinical decision making. EC not in cluster A, with poorer outcomes, exhibited lower Ki67 expression.

AFP Alpha-fetoprotein, *β- hCG* Human chorionic gonadotropin β, *CH* Choriocarcinoma, *CT* Chemotherapy, *EC* Embryonal Carcinoma, *IHC* immunohistochemistry, *IQR* Interquartile Range, *LDH* Lactate dehydrogenase, *RT* Radiotherapy, *SE* Seminoma, *TE* Teratoma, *YST* Yolk-sac Tumor.
