# Supplementary figures and images for: Ki67 and LSD1 Expression in Testicular Germ Cell Tumors Is Not Associated with Patient Outcome: Investigation Using a Digital Pathology Algorithm
Source: Life (Basel). 2022 Feb 10;12(2):264. doi: 10.3390/life12020264 (PMC8875543; doi:10.3390/life12020264)

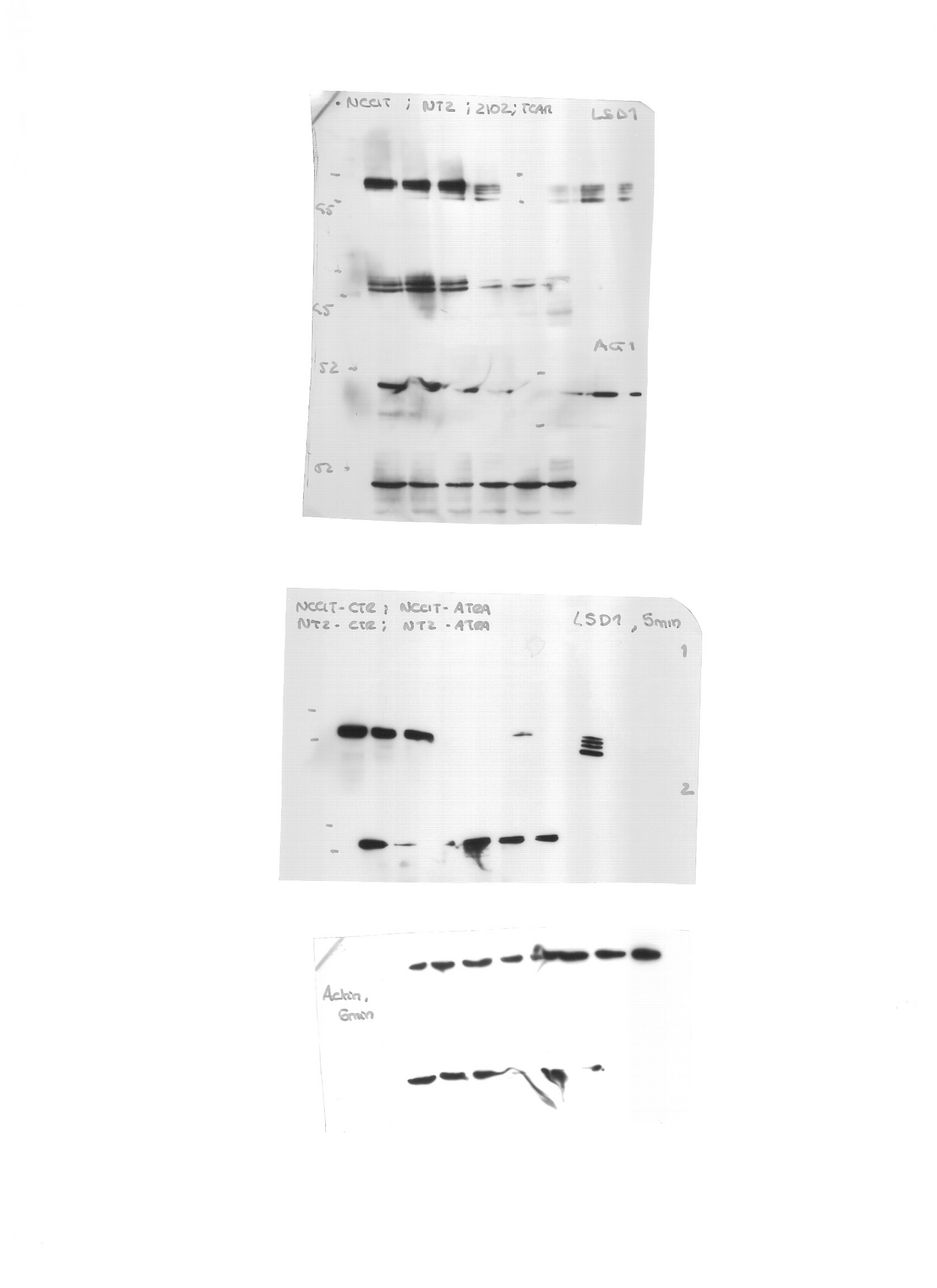

Supplement: Supplementary file 1 [file life-12-00264-s001.zip › life-12-00264-s001/Supplementary material - original blots.tif]
